# Attrition in cardiac surgery: Structural drivers, career pathways, and implications for workforce retention

**DOI:** 10.1016/j.xjon.2026.101981

**Published:** 2026-07-31

**Authors:** Iris Koch, Katharina Huenges, Tamari Tvildiani, Elpiniki Katsari, Ayse Cetinkaya, Nadejda Monsefi, Bastian Schmack, Anna L. Meyer, Jan D. Schmitto, Arjang Ruhparwar, Julie Cleuziou, Hassina Baraki, Gloria Faerber, Sabine Bleiziffer, Jasmin S. Hanke

**Affiliations:** a Department of Cardiothoracic, Transplantation and Vascular Surgery, Hannover Medical School, Hannover, Germany; b Clinic for Cardiac Surgery, University Hospital Schleswig-Holstein Campus Kiel, Kiel, Germany; c Department of Cardiac Surgery, Hospital Kassel, Kassel, Germany; d Office of the Medical Director, University of Greifswald, Greifswald, Germany; e Department of Cardiac Surgery, Kerckhoff-Heart Center Bad Nauheim, Justus-Liebig University Giessen, Bad Nauheim, Germany; f Department of Cardiac Surgery, University Hospital Bonn, Bonn, Germany; g Department of Cardiac Surgery, Heidelberg University Hospital, Heidelberg, Germany; h Department of Congenital and Paediatric Heart Surgery, German Heart Center, TUM University Hospital, Munich, Germany; i Department of Cardiac Surgery, University Hospital Basel, Basel, Switzerland; j Department of Thoracic, Cardiac, and Thoracic Vascular Surgery at the University Hospital Würzburg, Würzburg, Germany; k Department of Cardiac Surgery, Central Hospital Bad Berka, Bad Berka, Germany

**Keywords:** cardiac surgery, attrition, surgical training, residency, workforce retention, surgical education, management

## Abstract

**Objectives:**

Workforce shortages in cardiac surgery (CS) are increasing despite high initial interest among young physicians. Attrition remains poorly understood and is often attributed to individual limitations rather than systemic factors. This study aimed to identify determinants of attrition, characterize subsequent career pathways, and define targets for improving retention.

**Methods:**

A German nationwide, anonymous, questionnaire-based survey (47 items) was conducted between July 2024 and January 2026 among physicians with previous employment in German CS departments. Descriptive statistics and subgroup analyses were performed.

**Results:**

Seventy-five participants were included. Initial motivation for pursuing CS was high and mainly driven by interest in the specialty's technical and intellectual complexity. However, satisfaction with key aspects of training was low, particularly regarding surgical training (23.3% [17 out of 73]), scientific support (24.7% [18 out of 73]), and professional mentorship and career development (28.8% [21 out of 73]). The most frequently reported reasons for leaving were poor work-life balance (74.2% [46 out of 62]), dissatisfaction with workplace culture (67.7% [42 out of 62]), limited career opportunities (66.1% [41 out of 62]), and lack of appreciation (62.9% [39 out of 62]). Attrition occurred across all career stages, including board-certified surgeons and senior staff. Despite leaving the specialty, most participants were satisfied with their decision (95.1% [58 out of 61]), although only 11.7% (7 out of 60) considered returning to CS.

**Conclusions:**

This exploratory survey suggests that attrition from CS is frequently associated with dissatisfaction regarding work-life balance, workplace culture, career perspectives, and training conditions rather than with self-perceived individual limitations. Although causal relationships cannot be established, the findings highlight potentially modifiable organizational factors that may represent targets for future retention strategies in CS.

**Figure:**
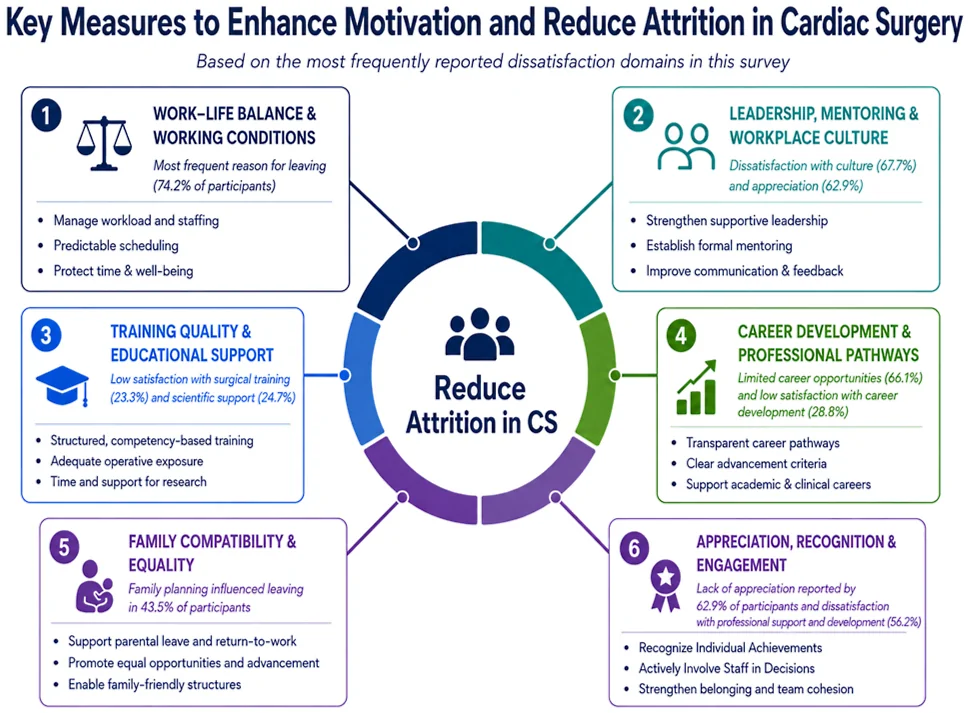
Key measures to reduce attrition in cardiac surgery.

**Central Message**

This study suggests attrition from cardiac surgery is linked more to work-life balance, workplace culture, career prospects, and training than to individual limitations.

**Perspective**

Cardiac surgery continues to attract highly motivated physicians, yet many leave the specialty due to structural rather than individual factors. This study highlights the central role of workplace culture, training quality, mentorship, career transparency, and work-life balance in attrition and emphasizes the need for sustainable retention strategies to secure the future workforce.

Cardiac surgery (CS) is renowned for its innovation, complex procedures, and interdisciplinary research opportunities.^1^^,^^2^ Despite its scientific and technical appeal, the specialty faces increasing challenges in recruiting and retaining qualified physicians. Although interest among medical students and junior doctors remains high, many trainees but also more experienced surgeons leave the field at different career stages, posing a possible threat to workforce sustainability.^3^ CS departments operate under demanding conditions shaped by economic and regulatory pressures, high (on-call) workload, increasing patient complexity, and intergenerational differences in workplace expectations. Consequently, management must not only attract talented junior staff but moreover retain and motivate experienced specialists.^3^, ^4^, ^5^

Attrition in CS has significant consequences: The loss of trained surgeons represents a major waste of resources and expertise, whereas high turnover may negatively influence team stability, continuity of care, and future academic leadership. Previous studies in nonmedical fields have linked attrition to demanding working conditions, limited flexibility, and restricted career progression.^6^, ^7^, ^8^ However, attrition in CS has not been sufficiently studied.

Little is known about why physicians leave CS, when these decisions occur, and which career paths they pursue afterward. Especially structural factors such as training quality, mentorship, workplace culture, and career transparency remain unsatisfactorily investigated. This lack of systematic data limits the development of effective retention strategies. Therefore, this study aimed to analyze attrition in cardiac surgery by identifying structural drivers, examining postexit career pathways, and defining targets to improve long-term retention.

## Material and Methods

### Ethical Statement

Participation in the anonymous survey was voluntary. Before completing the survey, all participants were informed about the purpose of the study and the planned publication of the results and provided informed consent to participate. Written informed consent was not obtained because the survey was anonymous and participation itself indicated consent. Ethical approval was not required because the study was based exclusively on anonymous and aggregated data. No identifiable participant information was collected or published. Accordingly, individual informed consent was not required under institutional and local ethical requirements.

### Survey Design

A nationwide, anonymous, questionnaire-based survey was conducted between July 2024 and January 2026 among physicians with prior employment in CS departments in Germany. Eligibility required a previous formal employment contract in a German CS unit. Individuals with ongoing employment in CS or without prior formal employment were excluded. The questionnaire consisted of 47 items covering demographic characteristics, career trajectories, training experiences, and reasons for leaving CS. The survey instrument was developed by the authors based on a review of the existing literature and expert consensus within the field of CS. Before distribution, the questionnaire was piloted with a small group of experienced cardiac surgeons to assess clarity and content validity.

The survey was distributed through professional networks, including the German Society for Thoracic and Cardiovascular Surgery, institutional contacts, LinkedIn, and other career-oriented online platforms. Participation was voluntary and anonymous. Because the survey was disseminated through professional networks and could be shared further among potential participants a response rate could not be calculated. To minimize duplicate entries, internet protocol address tracking was implemented. No financial incentives were provided. The anonymous study design was chosen to facilitate participation and encourage candid responses regarding potentially sensitive career decisions. Due to the anonymous online concept of the design and thus to ensure data quality and consistency, only completed questionnaires that met the inclusion criteria were included in the final analysis.

### Statistical Analysis

Data analysis was primarily descriptive, including frequencies, proportions, and measures of central tendency. Subgroup analyses were performed to explore potential differences between early and late career dropouts, as well as between male and female participants. Given the exploratory nature of the study and the sample size, nonparametric statistical tests (Mann-Whitney *U* test) were used where appropriate. These methods were selected due to their robustness in analyzing ordinal data and distributions that may deviate from normality. All analyses were conducted using SPSS IBM Statistics for Windows (version 29.0.1.0) (IBM-Germany). Due to the exploratory design, no formal sample size calculation was performed.

## Results

### Baseline Demographics

A total of 172 individuals participated in the survey, of whom 75 met the inclusion criteria and were included in the final analysis (43.6%). Reason for exclusion of the participants was incomplete submissions. The study population comprised 46.7% (28 out of 60) female and 53.3% (32 out of 60) male participants. Fifteen persons did not define their gender. Baseline demographics of the study cohort are displayed in Table 1.

**Table 1:** Baseline demographics of the study cohort

	%	n	Mean ± SD
No. of participants	100	75	
Sex distribution	100	60	
Male	53.3	32	
Female	46.7	28	
Average age at beginning CS (y)		75	27.9 ± 3.0
Average age at exit from CS (y)		74	36.6 ± 7.8
Focus on a specialist area during or after training	100	31	
Aortic surgery	16.1	5	
Heart valve surgery	16.1	5	
Congenital surgery	16.1	5	
Transplant surgery	12.9	4	
Heart failure surgery	9.7	3	
Coronary surgery	9.7	3	
Cardiac intensive/critical care	6.5	2	
Interventional procedures	6.5	2	
Arrhythymia surgery	6.5	2	
Last professional position before leaving CS	100	62	
Resident in training for CS	54.8	34	
Certified specialist in CS	19.4	12	
Consultant in CS	9.7	6	
Senior consultant for CS	14.5	9	
Head consultant for CS	1.6	1	
Highest academic qualification achieved	100	60	
Doctor without a medical doctorate∗	21.7	13	
Medical doctorate	66.7	40	
University lecturer/Assistant Lecturer	6.7	4	
Associate professor	3.3	2	
Tenured professor	1.7	1	
Career break	100	74	
No break (working full time)	83.8	62	
Career break	16.2	12	
Not achieving board certification	56.2	41	
Years of training when dropping out of CS (n = 38 responses)			3.5 ± 1.5
Not regretting the invested efforts (n = 35 responses)	88.6	31	
Achieving board certification	43.8	32	
Years when changing career after board certification (n = 28 responses)			4.9 ± 3.8
Not regretting the invested efforts (n = 29 responses)	79.3	23	

*CS*, Cardiac surgery.

∗ In Germany, the title Dr med is a separate doctoral qualification obtained after medical school and is not required for clinical practice.

Most participants left CS during early career stages, with 54.8% (34 out of 62) working as residents at the time of exit. However, attrition was observed across all career levels, including certified specialists (19.4% [12 out of 62]) and senior positions (25.8% [16 out of 62]). Information on career breaks during training was available for 74 participants. The majority worked continuously in full-time positions (83.8% [62 out of 74]), whereas 16.2% (12 out of 74) reported a temporary interruption. Most cases for temporary interruption were related to parental leave (66.7% [8 out of 12]), predominantly among female participants (female 87.5% [7 out of 8])/male 12.5% [1 out of 8]), whereas a smaller proportion reported sabbaticals (8.3% [1 out of 12]) or other reasons (25.0% [3 out of 12]). The duration of leave was typically between 7 and 12 months. Only a minority of participants indicated that the career break contributed to their decision to leave cardiac surgery.

Among the participants 56.2% (41 out of 73) did not achieve board certification. The most frequently reported reasons were early transition to another specialty, an unfavorable training sequence, and insufficient supervisory support. On average, participants discontinued training after 3.5 years. Despite not completing board certification, the majority (88.6% [31 out of 35]) did not regret their investment in training (Table 1). In contrast, 43.8% (32 out of 73) of participants had completed board certification before leaving CS. These individuals changed careers after a mean of 4.9 years following certification. Only a minority (20.7% [6 out of 29]) reported regret regarding their training efforts (Table 1).

### Decision to Pursue a Career in CS and Further Development

Initial motivation for pursuing a career in CS among the participants was primarily driven by interest in the heart (57.3% [43 out of 75]), the technical complexity of the field and the perceived intellectual challenge (57.3% [43 out of 75]) as well as inspiration by role models (38.7% [29 out of 75]). Factors such as structured training (76.0% [57 out of 75]), mentorship (80.0% [60 out of 75]), inspiring mentors (74.7% [56 out of 75)] and, above all, a fascinating occupation (93.3% [70 out of 75]) and high professional standards (96.0% [72 out of 75]) were rated as highly important at career entry, whereas work-life balance (25.3% [19 out of 75]) or regulated working hours (26.7% [20 out of 75]) played only a minor role at this career stage (Figure 1).

**Figure 1:**
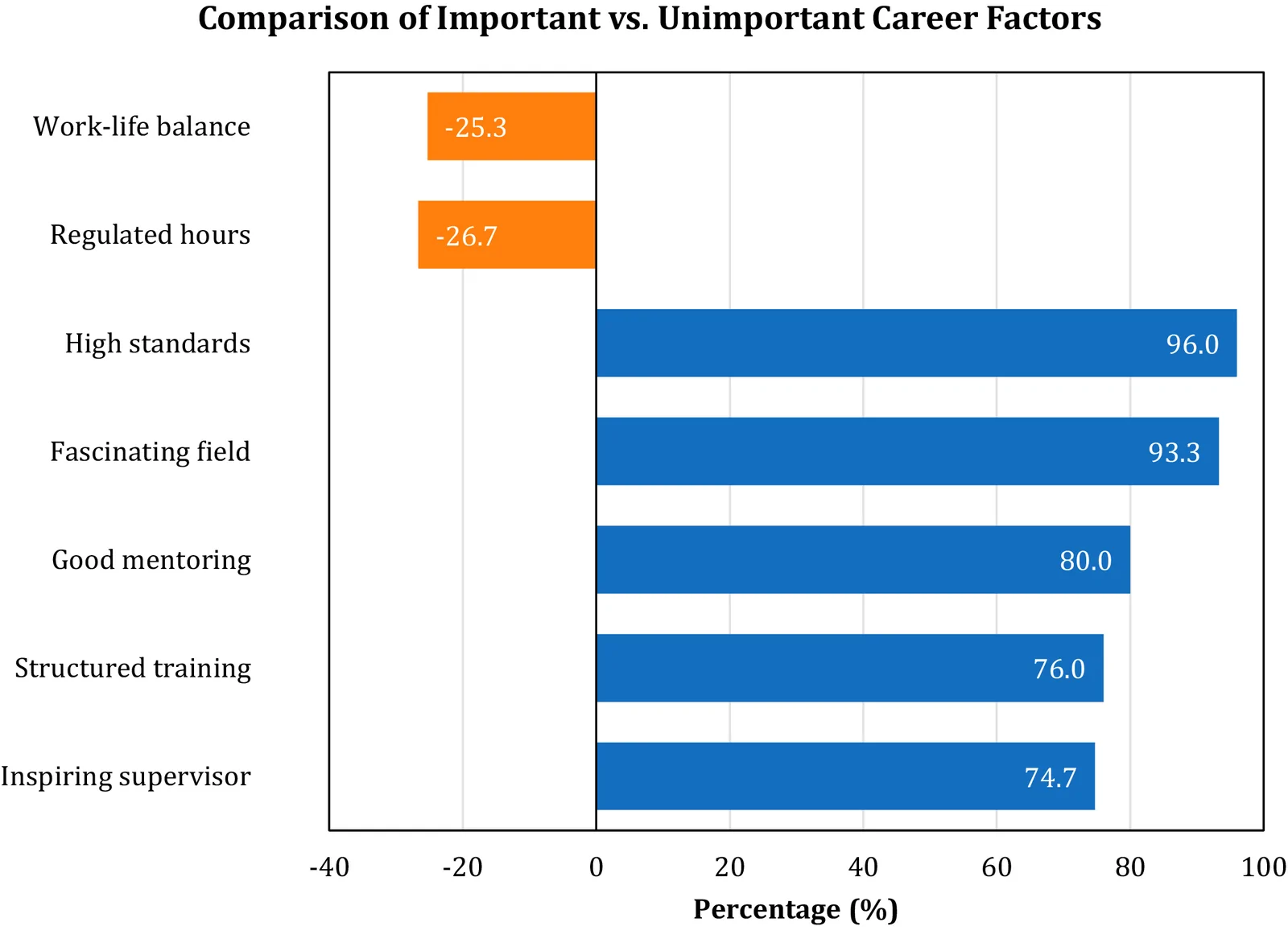
Comparison of important versus unimportant career factors at career entry among respondents (% respondents with attributed importance or insignificance is given in each case). Positive values represent factors rated as important, whereas negative values represent factors rated as unimportant.

At career entry, most participants reported specific professional goals. A total of 38.7% (29 out of 75) aimed for senior or head consultant positions, 28.0% (21 out of 75) for board certification as a cardiac surgeon, and 14.7% (11 out of 75) for a chief cardiac surgeon role. In contrast, 16.0% (12 out of 75) reported no specific career objectives, whereas 2.7% (2 out of 75) indicated alternative goals such as academic qualification.

### Satisfaction With Training in CS

Participants rated their satisfaction with different aspects of training on a 5-point Likert scale. Responses were available for 73 participants. Overall satisfaction with training components was low. Only 28.8% (21 out of 73) of participants reported satisfaction with professional support and career development, 23.3% (17 out of 73) with surgical training, and 24.7% (18 out of 73) with scientific training. In contrast, satisfaction with clinical training was higher, with 53.4% (39 out of 73) of participants reporting positive ratings. Overall, with the exception of clinical training, most participants expressed dissatisfaction with key components of training in CS (Figure 2).

**Figure 2:**
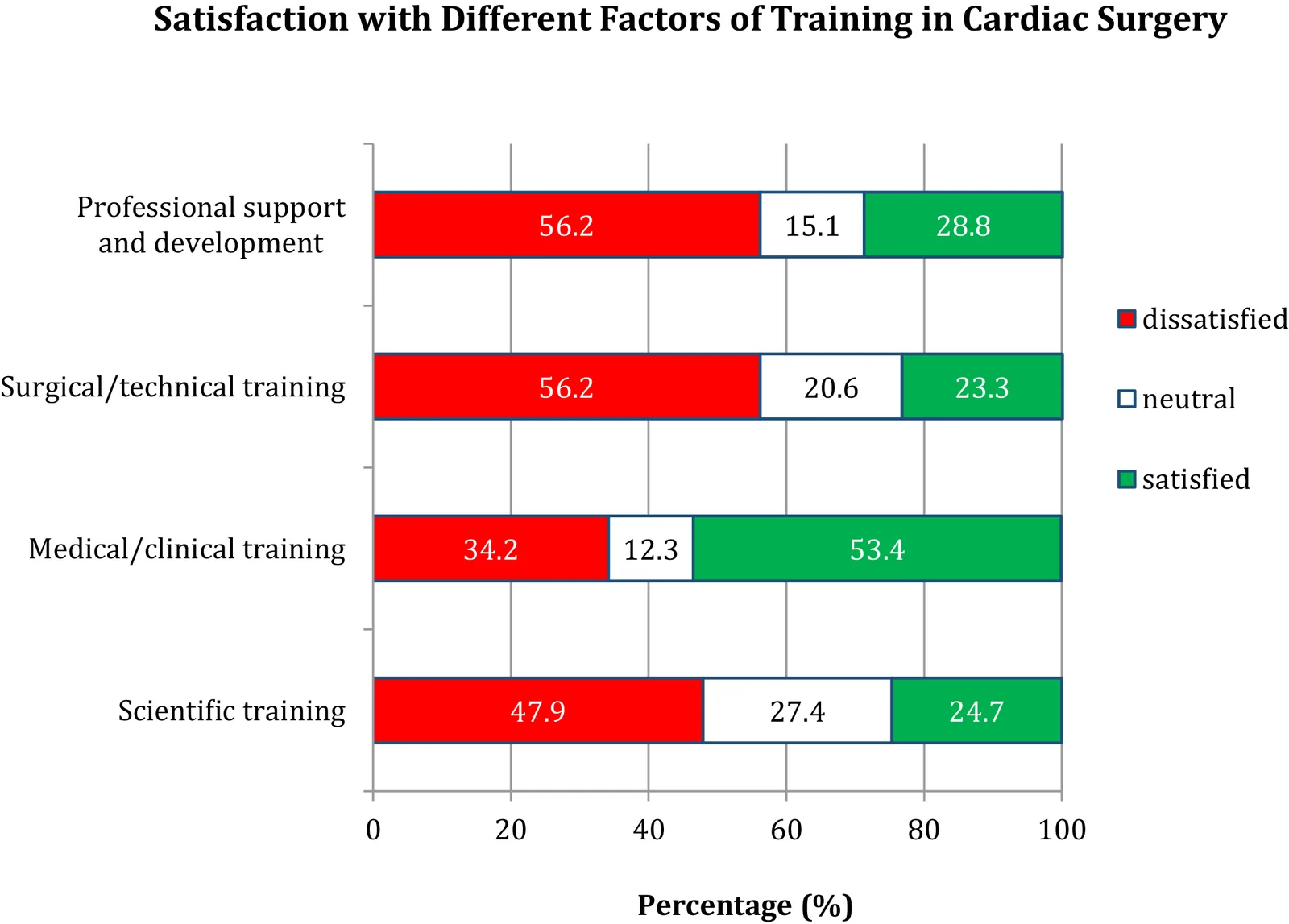
Satisfaction with different factors of training in cardiac surgery (% respondents and their attributed degrees of satisfaction/dissatisfaction for each factor).

### Reasons for Ending a Career in CS

Data on the time interval between the intention to leave and actual departure were available for 64 participants. In the majority of cases, the decision was implemented within a relatively short period: 39.1% (25 out of 64) left within <6 months, and a further 20.3% (13 out of 64) within 1 year. Longer intervals were less common, with 21.9% (14 out of 64) reporting 1 to 2 years and 18.8% (12 out of 64) more than 2 years. Information on discussions with supervisors or mentors was available for 62 participants. Of these, 58.1% (36 out of 62) reported having discussed their intention to leave before their departure.

Participants rated the relevance of different factors contributing to their decision to leave CS. Responses were available for 62 participants. Structural and organizational factors were identified as the primary drivers of attrition. The most frequently reported reasons were poor work-life balance (74.2% [46 out of 62]), dissatisfaction with workplace culture (67.7% [42 out of 62]), the availability of more attractive career alternatives (67.7% [42 out of 62]), limited career opportunities (66.1% [41 out of 62]), and lack of appreciation (62.9% [39 out of 62]) (Figure 3).

**Figure 3:**
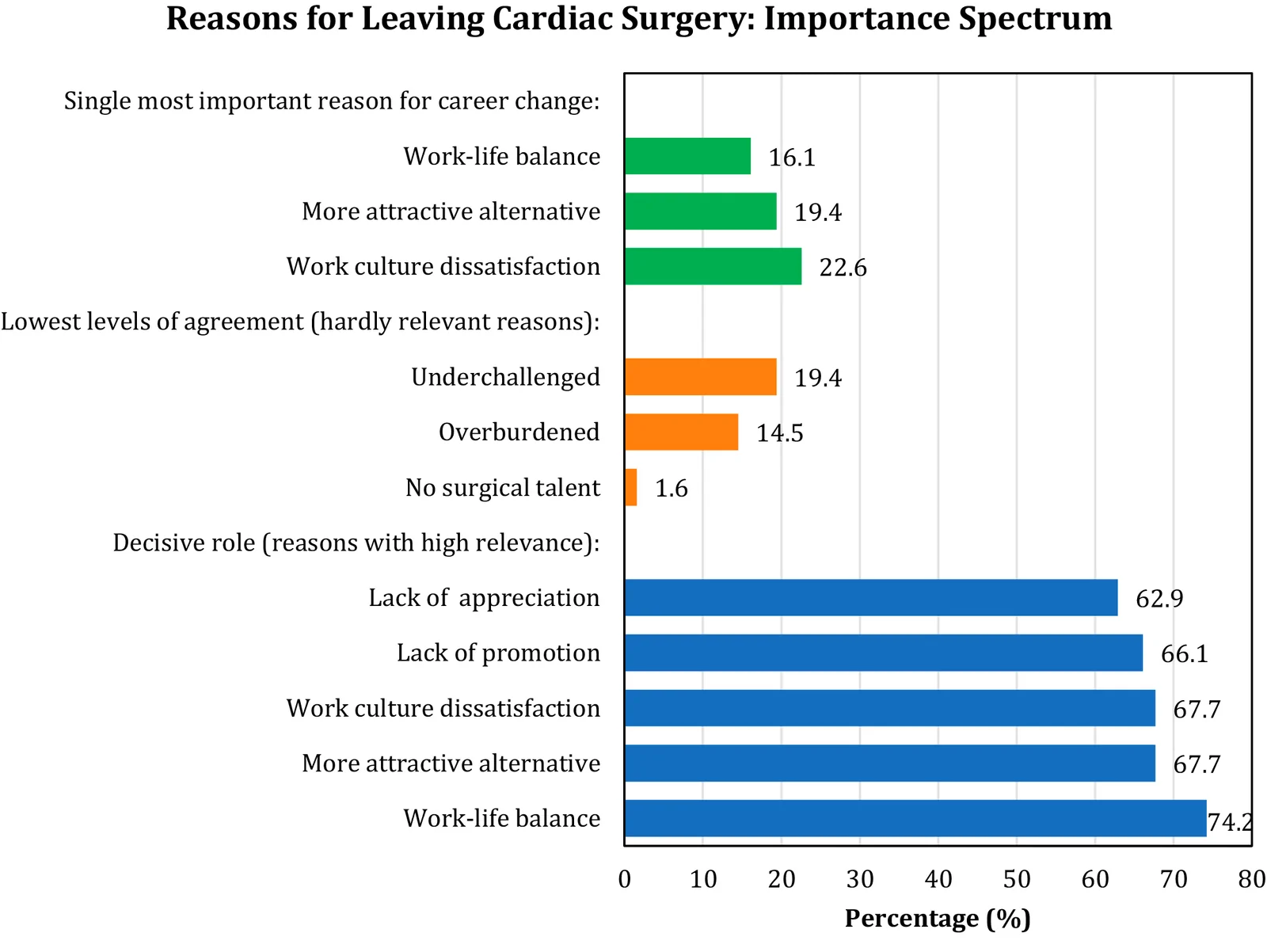
Most common reasons for ending a career in cardiac surgery (% respondents with attributed relevance for each case).

In contrast, individual factors played only a minor role. Very few participants attributed their decision to a lack of surgical talent (1.6% [1 out of 62]), feeling overburdened (14.5% [9 out of 62]), or being underchallenged (19.4% [12 out of 62]) (Figure 3). When asked about the single most important reason for a career change 14 out of 62 surveyed persons (22.6%) chose the answer “dissatisfaction with the work culture in general (no open feedback/poor communication, more ‘blaming’ for work deficiencies, bad atmosphere in the team)”, followed by 19.4% (12 out of 62) with the answer “more interesting professional alternative” and 16.1% (10 out of 62) with the answer “work-life balance” (Figure 3).

Information on the influence of family planning was available for 62 participants. Overall, 43.5% (27 out of 62) reported that family planning contributed to their decision to leave CS (male, 51.9% [14 out of 27] and female, 48.1% [13 out of 27]), with 22.2% (6 out of 27) describing this influence as very strong (male, 33.3% [2 out of 6]) and female, 66.7% [4 out of 6]). Conflict-related reasons were less frequently reported. A total of 21.0% (13 out of 62) of participants indicated that they left CS due to a conflict situation, most commonly involving the chief surgeon (46.2% [6 out of 13] of this subgroup). Retrospective assessment of career expectations was available for 61 participants. A total of 26.2% (16 out of 61) reported that they had already anticipated early in their career that they would not remain in CS, whereas for the majority (73.8%, [45 out of 61]) such a development had not been foreseeable (Figure 4, *A*).

**Figure 4:**
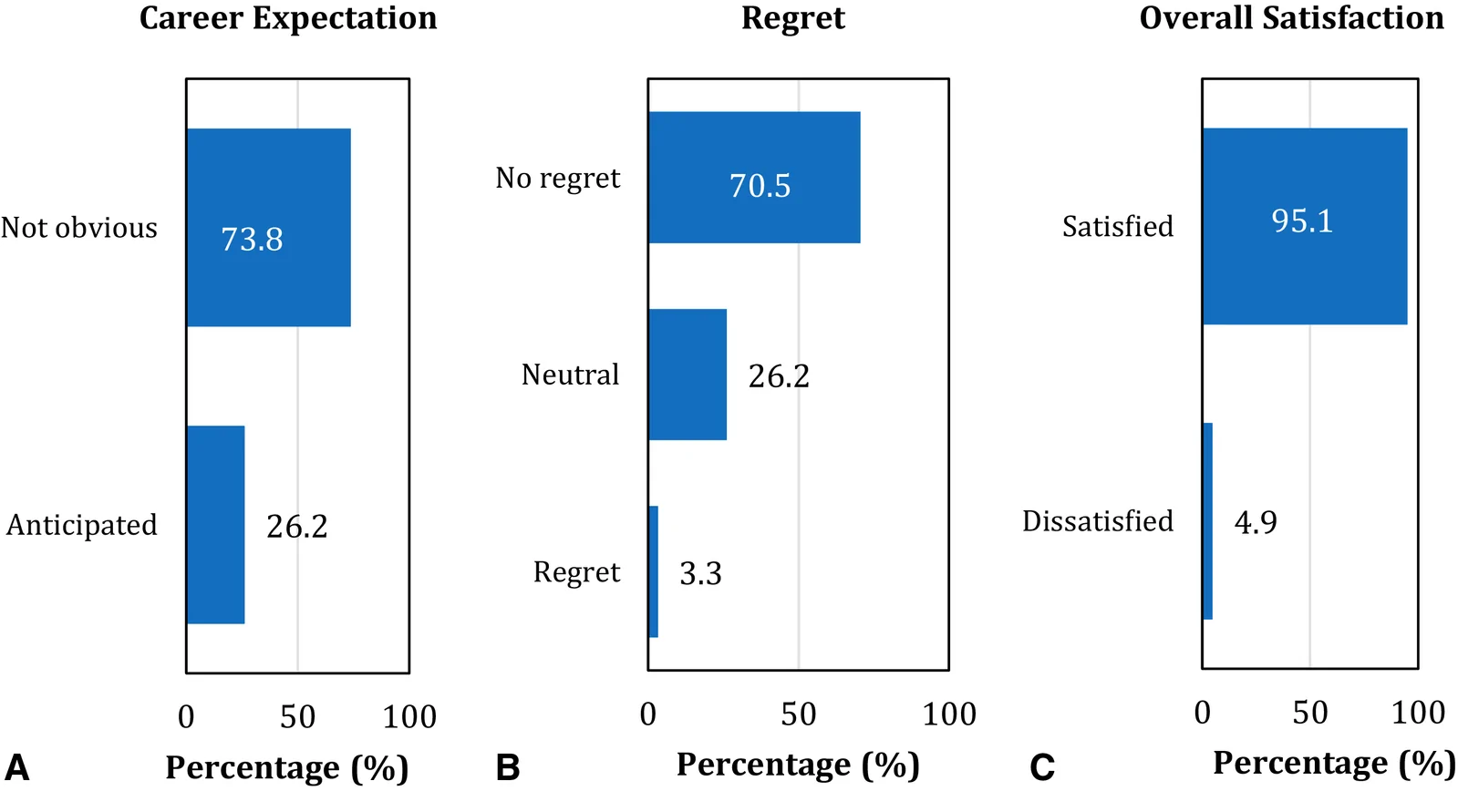
Career expectation and anticipation of leaving cardiac surgery (*CS*), possible regret, and overall satisfaction with the decision to leave CS (% respondents).

Despite leaving the specialty, overall satisfaction with the decision to leave CS was high. Most participants (70.5% [43 out of 61]) reported no regret and described themselves as rather or very satisfied, whereas 26.2% (16 out of 61) were neutral and only 3.3% (2 out of 61) expressed regret (Figure 4, *B*). Notably, the overwhelming majority of participants did not regret leaving cardiac surgery (95.1% [58 out of 61]) (Figure 4, *C*).

### New Career Perspectives

Current professional fields were reported by 61 participants, with multiple responses permitted (73 responses in total). The most common career destinations were general medicine and cardiology (Figure 5). Compared with their previous position in cardiac surgery, 54.1% (33 out of 61) of participants reported improved salary in their current role, whereas 45.9% (28 out of 61) reported no improvement or a decrease. Key factors influencing career satisfaction in the new field included flexibility (73.8% [45 out of 61]), career advancement opportunities (70.5% [43 out of 61]), and leadership prospects (60.7% [37 out of 61]). In contrast, incentives such as childcare support (63.9% [39 out of 61]), employer benefit programs (62.3% [38 out of 61]), and home office options (60.7% [37 out of 61]) were rated as less important. Likelihood of returning to CS was assessed in 60 participants. The majority (73.3% [44 out of 60]) reported that a return would be unlikely, whereas only 11.7% (7 out of 60) considered it likely; 15.0% (9 out of 60) were undecided.

**Figure 5:**
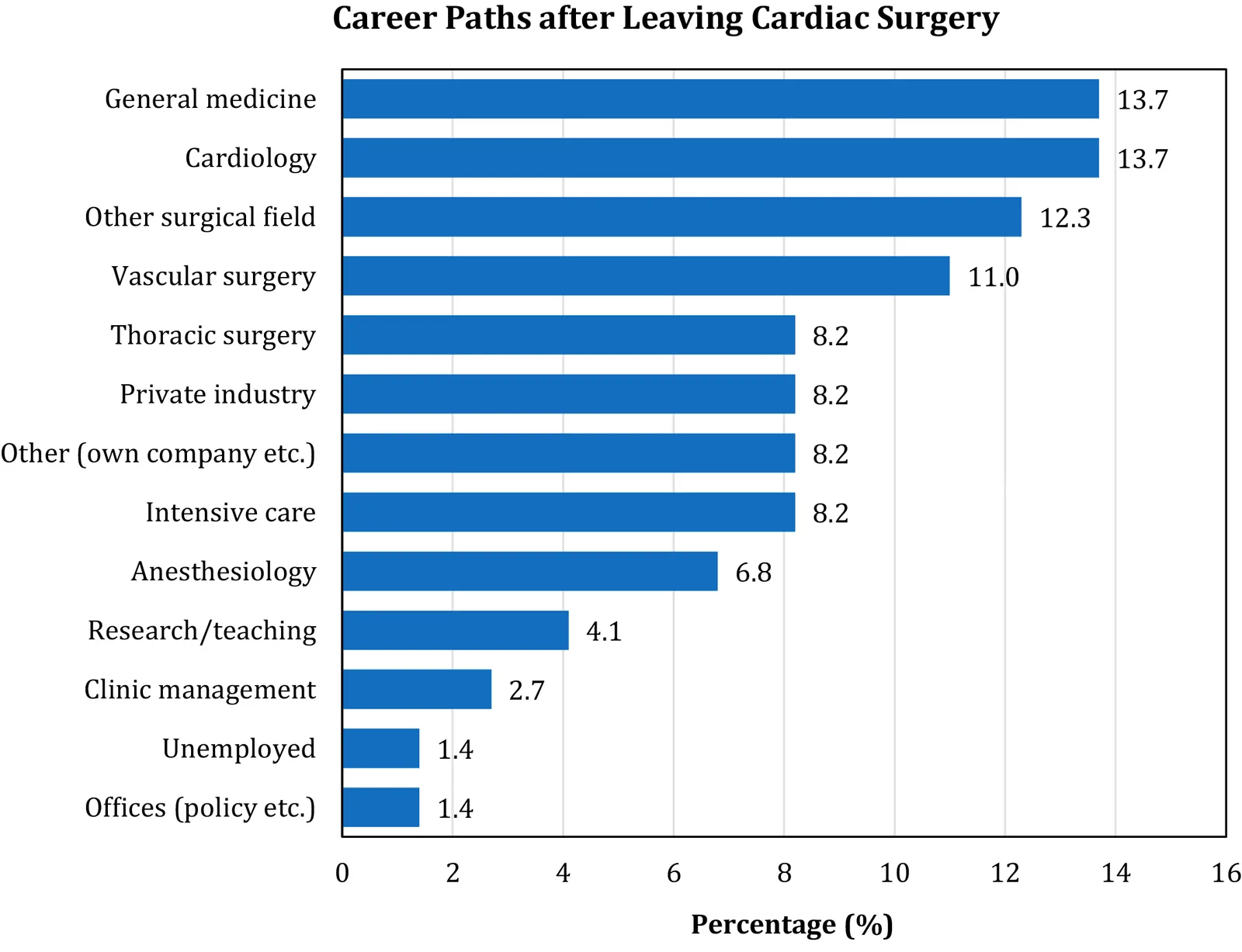
Changing career destinations after leaving cardiac surgery (% respondents).

Participants were also asked about potential reasons for a hypothetical return. Multiple responses were permitted (112 responses from 60 participants). Continued professional interest in CS was the most frequently cited reason (68.3% [41 out of 60]), followed by missing surgical activity (46.7% [28 out of 60]). In contrast, very few participants indicated that leaving cardiac surgery had been a mistake (1.7% [1 out of 60]) or that recovery of health would enable a return (3.3% [2 out of 60]).

### Suggestions for Improvements of Working Conditions in CS

At the end of the survey, participants were given the opportunity to provide free-text suggestions for improvements in CS. A total of 36 out of 75 respondents (48.0%) made use of this option. The responses revealed a heterogeneous pattern of feedback. Many participants addressed multiple areas simultaneously rather than focusing on a single aspect. Overall, at least 4 key areas for improvement were identified, with some thematic overlap between them:

• Training and continuous professional development, mentioned by 50.0% (18 out of 36) of respondents who provided free-text feedback.
• Working conditions, including work-life balance, general career development and promotion, as well as the advancement of women, mentioned by 44.4% (16 out of 36).
• Leadership, communication, and feedback culture, including interpersonal relationships and team cohesion, mentioned by 33.3% (12 out of 36).
• Structural changes, such as increasing practical training and improving teaching within departments, mentioned by 27.8% (10 out of 36).

## Discussion

Neither absolute nor temporal trends in attrition of the CS workforce can be reliably determined due to the lack of robust national or international data. This study provides the first analytical insights into the determinants of attrition from cardiac surgery and demonstrates that workforce fluctuation in this specialty is predominantly shaped by structural rather than individual factors.

Across all analyses, attrition was consistently associated with a cluster of structural deficiencies, particularly insufficiently structured training, limited career transparency, and an unsupportive work environment. Dissatisfaction with surgical and scientific training, as well as inadequate mentorship and feedback culture, were frequently reported and appear to play a central role in shaping career decisions. In later stages, work-life imbalance further contributed to the decision to leave.

The lack of standardized educational structures across German CS training programs may contribute to workforce instability.^9^^,^^10^ In many institutions, career progression remains informal, nontransparent, and influenced by local hierarchies. This may foster perceptions that advancement depends on other aspects than objective performance criteria or structured planning, potentially discouraging open communication, feedback-seeking, and early identification of training deficits.^11^ Over time, this may limit professional development and job satisfaction.^10^^,^^12^^,^^13^

Although improved selection of trainees may contribute to improved retention, attrition is unlikely to be solved through stricter selection criteria alone. The factors identified in this study suggest that modifiable aspects of training quality, mentorship, workplace culture, and career development opportunities may play a more important role in long-term retention. A notable finding is that most participants did not leave procedural medicine altogether but transitioned into closely related specialties. This might suggest that neither surgical competence nor interest in complex interventions constitutes the primary limiting factor. Rather, CS appears to lose both early-career and experienced physicians to fields offering more predictable career trajectories, greater flexibility, and improved compatibility with personal life planning.^10^^,^^14^^,^^15^ The concentration of CS services in relatively few high-volume centers inherently restricts job availability, intensifies competition, and limits flexibility. All factors that are likely less evident in neighboring disciplines such as vascular, thoracic surgery, cardiology or anesthesiology. Although the traditionally competitive culture in CS may promote high performance and excellence, a more supportive environment with continuous emphasis on excellence could improve well-being, psychological safety, and long-term commitment.^16^^,^^17^

The implications of these findings extend beyond individual career decisions. Given the extensive time and resources required for surgical training, attrition represents a significant loss at both institutional and economical levels. Moreover, the disproportionate loss of early-career surgeons may compromise the future pipeline of academic leaders and innovators.^10^^,^^13^^,^^18^

Although gender was not the primary focus of this study, women were overrepresented in our cohort compared with the reported proportion of women in CS in Germany (46.7% vs 25%). This may partly explain the relevance of family planning and age at exit observed in our results. Women appeared to leave the field earlier than men, potentially reflecting gender-specific career trajectories and family planning considerations. However, work-life balance and family-related factors were also relevant among male participants, suggesting that these challenges influence the broader cardiac surgical workforce rather than women alone.

Taken together, these findings indicate that attrition in CS is primarily a consequence of modifiable structural and cultural deficiencies rather than a lack of motivation, aptitude, or commitment among trainees and surgeons. Participants consistently identified deficiencies in training structure, mentorship, career transparency, and work-life integration as key factors influencing their decision to leave. Efforts to improve retention should therefore focus on reforming the training and working environment rather than on selecting different candidates or expecting greater individual resilience.^9^^,^^10^^,^^19^ Structured competency-based training pathways should become a standard component of CS education. Departments should provide transparent operative milestones, regular performance evaluations, protected mentorship, and clearly communicated career expectations. Reliance on informal hierarchies and opaque advancement processes risks creating uncertainty, reducing trust, and ultimately driving talented individuals away from the specialty.

Equally important, CS must adapt to changing professional expectations. The historical culture of long working hours and personal sacrifice may no longer be compatible with the priorities of many highly qualified physicians. If the specialty wishes to remain competitive in attracting and retaining talent, greater emphasis must be placed on work-life integration, flexible career models, family-friendly working conditions, and psychological safety. Excellence and sustainability should not be viewed as competing goals but as complementary requirements for a successful modern workforce.

The finding that most participants remained in procedural or closely related specialties is particularly noteworthy. CS is therefore not primarily losing individuals from medicine or surgery altogether; rather, it is losing them to disciplines perceived as offering better training experiences, more transparent career pathways, and more sustainable working conditions. This should be interpreted as a warning signal for training programs and professional societies. Without meaningful structural reforms, continued loss of highly qualified surgeons may threaten workforce stability, academic productivity, and future leadership within the specialty. Consequently, retention should be recognized as a strategic priority for departments, professional societies, and health care policymakers. Workforce planning must extend beyond operative demand and training capacity to include long-term career sustainability. Systematic collection of workforce and attrition data, routine exit interviews, transparent employment pathways, and targeted retention initiatives should become integral components of future workforce strategies.^9^^,^^19^, ^20^, ^21^

Future research should focus on longitudinal and multicenter designs to better understand career trajectories and identify critical time points for intervention. Comparative analyses across health care systems may further clarify how structural and cultural factors influence workforce stability in CS.^9^^,^^20^^,^^21^ Ultimately, preserving excellence in CS will require a cultural shift from reliance on individual endurance toward structured development, supportive leadership, transparency, and sustainable career models. Failure to address these issues risks continued loss of talented surgeons. On the contrary, successful reform offers the opportunity to strengthen workforce stability, innovation, education, and patient care for future generations. Figure 6 summarizes key measures that may support retention and reduce attrition in cardiac surgery.

**Figure 6:**
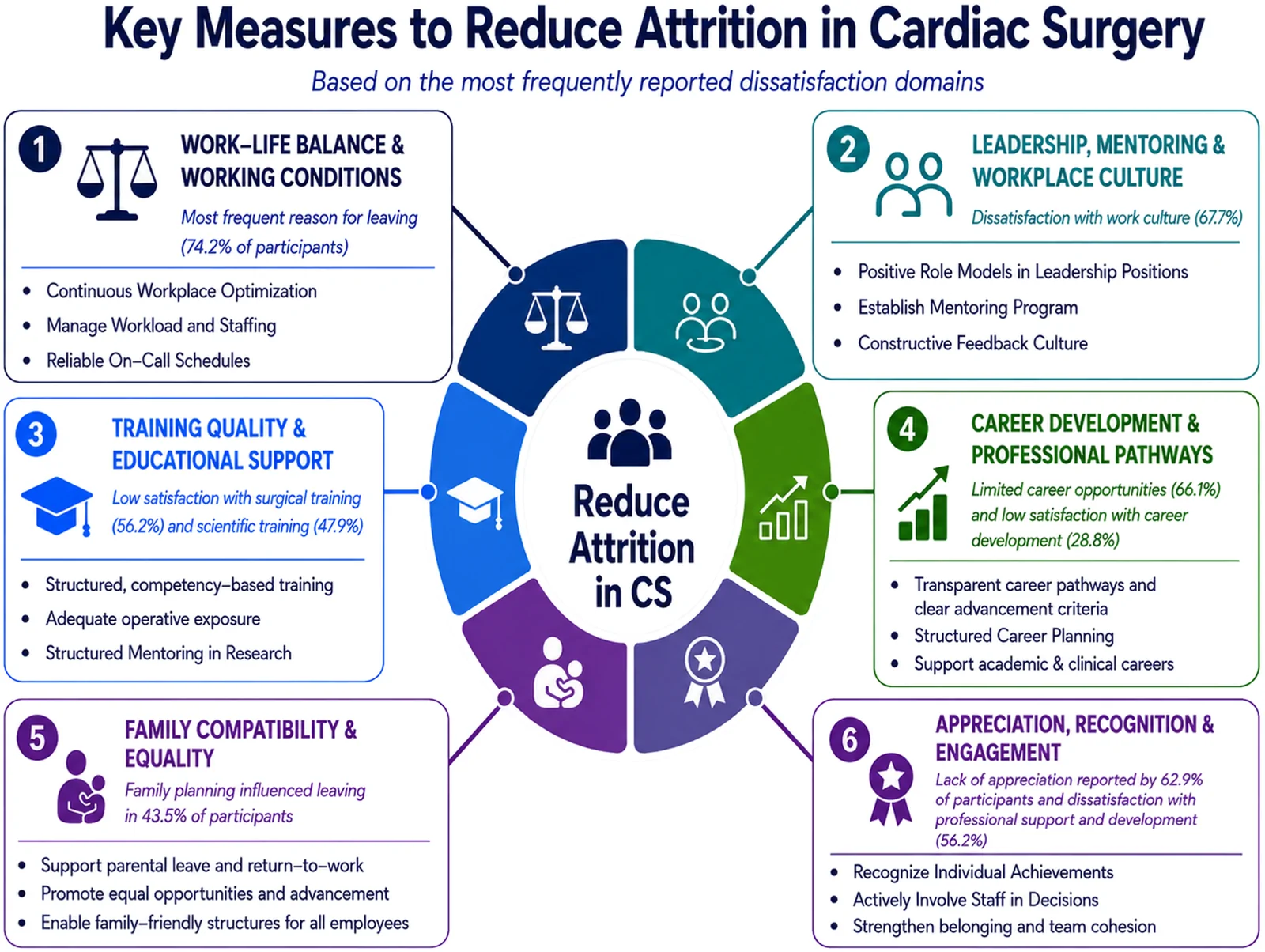
Key measures to reduce attrition in cardiac surgery based on this study's findings.

### Limitations

This study has several limitations. The online, anonymous survey design limited control over sampling and cannot fully exclude duplicate participation, despite internet protocol address tracking. Self-reported data may be influenced by recall, perception, and response bias, particularly regarding retrospective assessments of career decisions, skills, and workplace experiences. The cross-sectional design precludes causal inference. Therefore, findings should be interpreted as associative. In addition, the relatively small sample size, restriction to the German CS context, and exclusion of incomplete responses may limit generalizability. Finally, the study identifies structural and cultural factors associated with attrition but cannot quantify their relative influence or interactions. Future prospective, longitudinal studies are needed to validate these findings, identify critical intervention points, and evaluate targeted retention strategies.

## Conclusions

This first exploratory survey specifically addressing attrition from CS suggests that leaving the specialty is frequently associated with dissatisfaction regarding work-life balance, workplace culture, career perspectives, and training conditions rather than with (self-perceived) individual limitations. Although causal relationships cannot be established, these findings identify potentially modifiable organizational factors that may serve as targets for future retention strategies in CS.

### Declaration of Generative AI and AI-Assisted Technologies in the Writing Process

During preparation of this work the author(s) used ChatGPT for language editing. After using this tool, the, the author(s) reviewed and edited the content as needed and take(s) full responsibility for the content of the publication.

## Conflict of Interest Statement

The authors reported no conflicts of interest.

The *Journal* policy requires editors and reviewers to disclose conflicts of interest and to decline handling or reviewing manuscripts for which they may have a conflict of interest. The editors and reviewers of this article have no conflicts of interest.
